# The association between adverse childhood experiences and mental health problems in young offenders

**DOI:** 10.1007/s00787-020-01608-2

**Published:** 2020-08-02

**Authors:** Daniel Turner, Anne Jule Wolf, Steffen Barra, Marcus Müller, Priscilla Gregório Hertz, Michael Huss, Oliver Tüscher, Wolfgang Retz

**Affiliations:** 1 Department of Psychiatry and Psychotherapy, University Medical Center of the Johannes Gutenberg-University Mainz, Untere Zahlbacher Straße 8, 55131 Mainz, Germany; 2 Neurocenter - Institute for Forensic Psychology and Psychiatry, Saarland University Medical Center, Homburg, Germany; 3 Department of Child and Adolescent Psychiatry, University Medical Center of the Johannes Gutenberg-University Mainz, Mainz, Germany

**Keywords:** Emotional abuse, Physical abuse, Sexual abuse, ADHD, Intermittent explosive disorder

## Abstract

High rates of adverse childhood experiences (ACEs, e.g., abuse and neglect) have been found in young offenders. Furthermore, ACEs seem to increase the risk of developing relevant mental health problems, in non-offending juveniles and adults. However, this association has only seldomly been addressed in offending juveniles and young adults. The present study aimed at evaluating the prevalence of ACEs and mental health problems as well as their association within a sample of male and female young offenders. Altogether, 161 adolescent and young adult offenders (16.8% females) from the youth detention center Worms (Germany) filled out questionnaires concerning ACEs and mental health problems with a focus on attention-deficit/hyperactivity disorder and intermittent explosive disorder. Considerable rates of mental health problems were found, e.g., a prevalence of 35.9% was found for intermittent explosive disorder. Furthermore, a greater proportion of the female offenders fell into the clinically significant category for somatic complaints, anxiety/depression, and attention problems than the male offenders. Female young offenders also reported more frequently about all forms of ACEs compared to the male offenders. Latent class analysis defined three subtypes of young offenders depending on their individual ACE patterns: (1) low ACEs, (2) mainly neglectful ACEs, and (3) multiple ACEs. ACEs were significantly associated with the occurrence of both internalizing and externalizing mental health disturbances, with the multiple-ACE subtype being most likely to report about significant mental health problems. The results of the present study point towards the relevance to routinely assess ACEs in young offenders to identify possible precursors of mental health problems and of future criminal behaviors.

## Introduction

Adolescents and young adults who have come into contact with the criminal justice system are an especially vulnerable group and, thus, in need for increased attention of the scientific community as well as policy-making authorities. Studying the characteristics of juvenile offenders and their delinquent pathways serves to identify specific treatment needs of this population. Among the risk factors that could be modified by successful therapeutic interventions are mental health problems [1, 2]. Mental health problems in adolescent and young adult offenders do not only increase the risk of criminal recidivism but they are also associated with behavioral disturbances during imprisonment, risky sexual behaviors, substance abuse, suicide attempts, and many other negative outcomes [3–9]. Thus, addressing mental health needs by effective therapeutic interventions could not only reduce the rate of criminal behaviors but could also help the young offenders to live a more satisfying life.

Across different countries, prevalence rates of mental disorders between 45 and 90% as well as high rates of comorbidity have been reported in juvenile and young adult offenders [10, 11]. These prevalence rates are considerably higher than those found in non-offending juveniles and young adults [12, 13]. Usually, externalizing disorders like attention-deficit/hyperactivity disorder (ADHD), substance use disorders and conduct disorders are more prevalent than internalizing disorders in young offenders [11, 14]. Internalizing disorders like depression or post-traumatic stress disorder (PTSD) or the simultaneous occurrence of multiple mental disorders are more frequently reported in female than in male young offenders [14–16]. Among externalizing disorders, intermittent explosive disorder (IED) has so far received only little attention in populations of young offenders, although IED should—due to its definition: the pathological expression of reactive and impulsive aggression—be closely related to violent and criminal behaviors. In this context, a prevalence rate of 11.4% has been reported in a sample of 280 young delinquent males from China [17]. To our knowledge, the prevalence of IED in young offenders from Western countries has not been evaluated so far.

The etiology of mental disorders is a multi-factorial process including developmental, social, personality, environmental and neurobiological factors. One risk factor closely associated with the development of mental disorders is adverse childhood experiences (ACEs), e.g., emotional, physical and sexual abuse or neglect [18–20]. ACEs are reported more frequently by young offenders compared to their non-offending counterparts and young offenders have been shown to be at elevated risk of poly-victimization, providing one possible explanation for the increased rate of mental disorders in young offenders [21, 22]. Moreover, ACEs also increase the risk for displaying early first-time and repeated criminal behaviors as well as criminal recidivism even over and above the risk increasing effect of mental disorders [23–25].

The association between ACEs, mental health, and criminal behaviors is addressed in the developmental psychopathology perspective [26]. The authors proposed that ACEs influence the biological and (neuro-)psychological development of individuals, for example, by causing chromosome damage or functional changes to the developing brain [27–29]. These developmental vulnerabilities in turn increase the likelihood to develop mental health problems, like depression, personality disorders, substance use disorders, or antisocial behaviors, which are known to be associated with offending and other maladaptive outcomes [26, 30, 31]. Comparable propositions can be found in the developmental taxonomy of Moffitt, which is still one of the most influential theories on juvenile offending [32]. The authors have identified two distinct trajectories of juvenile offending, the life-course persistent and adolescent-limited trajectory [32]. It was suggested that life-course persistent offenders or those individuals who start their criminal careers early in life and keep on offending during adulthood are more likely to have experienced ACEs which lead to a maladaptive neurobiological development resulting in neurocognitive problems [32, 33]. On a behavioral level, these neurocognitive deficits present themselves, for example, in an increased irritability, impulsivity, problems with emotion regulation and relationship problems [34, 35]. These behavioral disturbances again interact with the continuously persisting maladaptive and dysfunctional social environments, finally leading to the development of mental health problems like ADHD, oppositional defiant disorder, conduct disorder or other forms of pathological aggression [32, 36, 37]. Mental health problems in turn, just like in the developmental psychopathology perspective, increase the risk for future and on-going criminal behaviors [26, 32].

Despite its theoretical importance and despite the fact that a considerable number of studies have assessed the association between ACEs and mental health problems in non-offending juveniles, it was criticized that only very few studies so far have analyzed this connection in young offenders [38]. Most previous studies with young offenders had a rather narrow focus on specific forms of ACEs or specific psychiatric outcomes such as PTSD or substance use disorders [38, 39]. One of the few studies with a somewhat broader focus found that male juvenile offenders who had experienced emotional, physical and sexual abuse during childhood compared to juvenile offenders without any form of ACEs were more likely to be diagnosed with an affective disorder, an anxiety disorder, ADHD, PTSD, a substance use disorder or a disruptive behavior disorder [38]. Furthermore, those individuals who had experienced childhood physical, emotional and sexual abuse were more prone to re-arrests following the index offence compared to juvenile offenders without experiences of sexual abuse during childhood [38]. Furthermore, most studies have analyzed male offenders only and much less is known about female offenders.

Although current findings provide first evidence that childhood maltreatment is indeed associated with a broad range of mental health problems as well as re-offending in juvenile offenders, it cannot be neglected that only a small number of individuals who have experienced ACEs develop a mental disorder in later life or become criminals. It was suggested that a dose-dependent effect of cumulative ACEs could be responsible for this unique relationship [28]. Single forms of ACEs might not necessarily increase the risk of developing mental health problems, but it could rather be the accumulation of multiple, different forms of ACEs that is responsible for the occurrence of mental disorders. Thereby, each additional type of ACE seems to exponentially increase the risk of negative mental health outcomes [27, 28]. Furthermore, it was shown that juvenile and young adults who have experienced multiple types of ACEs show criminal behaviors in adolescence and during adulthood more frequently [22, 40]. Further research did not only highlight dose-dependent effects of cumulative ACEs on different outcomes in both offending and non-offending samples, but also pointed to the importance to consider specific ACE-related subtypes in this regard. This means that not only the number of ACEs but also their individually experienced patterns might be relevant to explain certain outcomes [41, 42].

The present study aims at providing further empirical evidence for the association between ACEs and mental health problems in young offenders. Thereby, our sample consists of male and female young offenders at the beginning of their criminal careers. Their evaluation appears particularly worthwhile to identify certain risk factors that, if addressed in treatment interventions, could prevent a chronic criminal lifestyle in young offenders. A wide range of mental health problems and other maladaptive behaviors, including anxiety, depression, somatic complaints, ADHD and IED are assessed within the present study, thereby adding important findings to the current state of research. In line with previous research, we hypothesized to find in our sample: (1) different subtypes of young offenders based on individually experienced ACE patterns, (2) high prevalence rates of mental disturbances, and (3) positive associations between the number of ACEs and the occurrence of mental disturbances. Since respective research including female young offenders is rare, we explored sex differences without specific a priori expectations [10, 38, 41].

## Methods

### Participants

In total, 161 adolescents or young adults (134 males, 27 females) with a mean age of 18.48 years (SD 2.1; range 14–25 years) were recruited for the present study. The participants had an average of 9.29 years of school education (SD 0.75; range 8–13 years) and 54 (33.8%) participants were still in school at the time of the study. Male and female participants did not differ concerning age or years in school. Robbery (*n *= 46; 27.7%), grievous bodily harm (*n *= 34; 20.5%), breach of narcotics law (*n *= 27; 16.2%), breach of school law/excessive school skipping (*n *= 22; 13.2%), excessive use of public transportation without a valid ticket (*n *= 16; 9.6%), driving without driver`s license (*n *= 9; 5.4%), libel (*n *= 4; 2.4%), fraud (*n *= 4; 2.4%), damage to property (*n *= 4; 2.4%) were the offences found within the present sample. Because some offenders were convicted for more than one offence, the total number exceeds 100%. On average, the study participants had to stay 2 weeks (SD 4.5 days) at the youth detention center.

### Procedure

The ethical review board of the Medical Council in Rhineland-Palatine (Germany) approved the study protocol of the present research. All participants were recruited at the youth detention center in Worms, Germany. In Germany, youth detention has to be differentiated from the juvenile prison service. While placement in the prison system is a clear form of punishment, the German law defines youth detention as an educational intervention for adolescents and young adults who have come into conflict with current laws for rather minor offences. Thereby, youth detention should help adolescents and young adults to cope with current personal and social difficulties by providing, e.g., social skills training, career guidance, and debt counselling. Young offenders can be placed in youth detention for a maximum of 30 days, whereby most juveniles have to stay at the detention center only on the weekend, so it does not interfere with school or job training.

Adolescents are usually informed by mail concerning the precise date their sentence at the youth detention center begins. Beginning in May 2018, all adolescents and young adults additionally received a detailed study information and a form of consent about 2–3 weeks prior to the start of their sentence by mail. The study information informed the participants that the study would consist of completing different questionnaires. Furthermore, it was pointed out that participation was completely voluntary, that consent could be withdrawn at any time, that all data were collected for scientific purposes only, would be saved pseudonymously first, and would be anonymized after data collection was completed. Participants were informed that whether or not they participated in the present study; this would not have any consequences on their stay at the youth detention center. In case a young offender was willing to participate, he or she was asked to bring the signed form of consent at the beginning of his or her sentence (legal guardians of participants younger than 18 years had to give their informed consent as well). Those with signed consent forms received the study questionnaires on the first day of their sentence at the youth detention center. Completed questionnaires were collected in a closed box. All participants were asked to fill out their questionnaires by themselves in their private rooms at the youth detention center. The only requirement for study inclusion was a minimum stay of 7 days.

### Questionnaires

#### Youth Self-Report (YSR) [43, 44]

The YSR is among the most widely used self-report scales concerning the assessment of psychopathological abnormalities in adolescents and young adults. The questionnaire consists of 103 items evaluating behavioral, emotional and physical complaints during the last 6 months. All items can be answered with either 0 = never; 1 = sometimes; 2 = always. The YSR consists of eight different symptom scales that are combined to three higher-order problem scales: (1) internalizing problems: withdrawn/depressed, somatic complaints, anxious/depressed; (2) externalizing problems: rule-breaking behavior, aggressive behavior; and (3) mixed problems: thought problems, attention problems, social problems. All raw values of the symptom scales can be transformed into standardized *T* values. The authors of the questionnaire have provided *T* value cut-offs to determine whether or not certain symptoms should be considered as clinically significant or not. The psychometric properties of the German version of the questionnaire can be considered as satisfying with Cronbach’s *α* between 0.57 and 0.86 for the subscales [44]. In this study, Cronbach’s *α* for the subscales was between 0.78 and 0.90.

#### Childhood Trauma Questionnaire-Short Form (CTQ-SF) [45]

The CTQ-SF is a 28-item version of the original CTQ [46] measuring ACEs experienced between the ages of 0 and 18 years on five domains: emotional abuse, emotional neglect, physical abuse, physical neglect, and sexual abuse. All items are rated on a 5-point Likert scale with response options ranging from “Never True” to “Very Often True”. Both the English and the German versions of the original CTQ and the CTQ-SF have yielded good psychometric properties; Cronbach’s *α* ≥ 0.82 for all subscales for the German version of the CTQ-SF except for physical neglect (Cronbach’s *α* = 0.53) [45, 47, 48]. In this study, Cronbach’s α for all subscales was between 0.64 (physical neglect) and 0.96 (emotional neglect).

#### Wender–Reimherr adult attention-deficit disorder scale self-report (WR-SR) [49, 50]

The WR-SR is a self-report scale consisting of 59 items evaluating ADHD symptoms based on the Utah Criteria. ADHD symptoms are assessed across ten different domains: attention difficulties, hyperactivity/restlessness, temper, affective lability, emotional over-reactivity, disorganization, impulsivity, oppositional symptoms, academic problems, and social attitude. All items are rated on a 5-point Likert scale ranging from 1 = “Not at all” to 5 = “Very much”. The scale has shown satisfactory psychometric properties (English version Cronbach’s *α* = 0.78; German version Cronbach’s *α* ≥ 0.83 for all subscales and Cronbach’s *α* = 0.98 for total score [49, 51]. In this study, Cronbach’s *α* for all subscales was between 0.72 and 0.90.

#### Intermittent Explosive Disorder-Screening Questionnaire for DSM-5 [52]

The DSM-5 diagnosis Intermittent Explosive Disorder (IED) refers to a condition of recurrent, problematic and impulsive aggression [53]. The first five items of the questionnaire (e.g., “Get into verbal fights or arguments with other people”) assess the frequency concerning certain aggressive behaviors on a six-point scale (0 = never happened; 5 = happened “so many” times that I can’t give a number), whereby a score of 12 and above qualifies the person for a possible IED diagnosis (IED total aggression score). The following seven items include questions regarding the frequency of verbal/nondestructive aggression, frequency of destructive aggression, proportionality of aggressive responses to provocation, impulsive or planned nature of aggressive outbursts, distress or impairment resulting from aggressive outbursts, and exclusionary factors related to the presence of psychiatric disorders, medication or drugs.

For the purpose of the present study, the English version of the questionnaire was translated into German by the first author. Back translation was performed independently by two persons with a master’s degree in psychology, both of whom were native German speakers, fluent in English, and blind to the original version of the IED-SF. Both back-translated versions were compared to the original English version by a third person who was an English native speaker and only slight differences between the original and the translated versions occurred. The found differences were resolved in group discussions of all persons involved in the translation process.

In the initial development and validation study of the English version of the questionnaire, good psychometric properties were found: concordance rate of *κ* = 0.80 with clinical diagnoses, positive predictive power of 0.96, negative predictive power of 0.86 [52]. In the present study Cronbach’s *α* was 0.80.

### Statistical analyses

Analyses were conducted in R and IBM SPSS version 26.0 for Mac [54]. To get an overview about the distribution of the included measures among the participants, we compared mean CTQ, YSR and WR-SR subscale scores as well as average lifetime aggression scores and the frequency of IED diagnoses between male and female participants. Beforehand though, YSR raw values were transformed into standardized *T* values. Because we were especially interested in psychopathological symptoms within our young offender group, we furthermore compared the relative number of male and female participants who reached a clinically significant symptom load across the single YSR subscales (*T* values above the predefined cutoff). ACEs were then considered in both person-centered and variable-centered approaches. Comparable to other studies, responses to the CTQ were dichotomized before analyses were performed: (1) Items with a score of 0 (“never true”) and 1 (“seldomly true”) were scored as absent and items with a score of 2 (“sometimes true”), 3 (“often true”), and 4 (“very often true”) were scored as present; (2) CTQ subscales were considered fulfilled if one respective item was scored as present [38].

To identify mutually exclusive subtypes of young offenders regarding their individual ACE patterns (person-centered approach), we performed Latent Class Analysis (LCA) using the poLCA function in R [55]. Based on the maximum likelihood calculations, young offenders were assigned to the specific latent class with the highest membership probability. Moreover, a CTQ-poly-victimization score (variable-centered approach) was built representing the cumulated number of different ACE categories by adding up the number of fulfilled CTQ subscales. To assess the associations between ACEs and mental disturbances, we compared the scores on YSR T-values, WR-SR and IED scales between ACE subtypes using MANOVAs and *χ*^2^ statistics. Furthermore, we conducted two different regression analyses for each YSR subscale score, for the WR-SR total score and the IED total score as outcome variables. In the first linear regression, the ACE subtypes were used as the predictor variables. In the second regression analysis, the CTQ-poly-victimization score was used as the predictor variable. In both linear regressions, age and sex were entered as covariates.

## Results

### Psychopathological symptoms and mean questionnaire scores

Comparisons of mean questionnaire scores between male and female participants are shown in Table 1. In total, the female participants reported higher frequencies on different ACEs compared to the male participants (emotional abuse, physical abuse, sexual abuse, and physical neglect). Furthermore, some psychopathological symptoms assessed with the YSR were also found more often among the female participants than the male participants (social problems, thought problems, attention problems, aggressive behaviors, externalizing problems) and female participants reported more often about symptoms related to ADHD.

**Table 1:** Average questionnaire scores and comparisons between female and male participants

	Total sample	Women (*n *= 27)	Men (*n *= 134)	*p*
*Childhood trauma*				
CTQ emotional abuse (max. 25)	10.01 (5.34)	13.81 (7.47)	9.20 (4.40)	< 0.01
CTQ physical abuse (max. 25)	8.61 (4.97)	10.70 (7.04)	8.17 (4.32)	0.02
CTQ sexual abuse (max. 25)	5.55 (1.94)	6.59 (3.26)	5.32 (1.45)	< 0.01
CTQ emotional neglect (max. 25)	14.25 (6.80)	13.37 (7.38)	14.44 (6.69)	0.46
CTQ physical neglect (max. 25)	10.79 (3.95)	12.04 (4.99)	10.53 (3.66)	0.07
CTQ-poly (max. 5)	2.43 (1.43)	2.67 (1.80)	2.38 (1.34)	0.34
*Psychopathological symptoms*				
YSR withdrawn/depressed	55.24 (7.80)	57.26 (9.80)	54.83 (7.56)	0.15
YSR somatic complaints (max. 80)	55.31 (7.31)	56.63 (8.95)	55.04 (6.95)	0.31
YSR anxious/depressed (max. 80)	56.29 (9.21)	58.11 (10.44)	55.93 (8.95)	0.26
YSR social problems (max. 80)	53.0 (6.06)	55.41 (8.57)	52.51 (5.33)	0.02
YSR thought problems (max. 80)	57.25 (8.64)	60.52 (8.44)	56.59 (8.56)	0.03
YSR attention problems (max. 80)	55.58 (8.40)	59.44 (11.65)	54.80 (7.39)	0.01
YSR rule-breaking behavior (max. 80)	58.24 (9.68)	60.85 (11.01)	57.72 (9.14)	0.13
YSR aggressive behavior (max. 80)	54.38 (7.29)	57.78 (9.14)	53.69 (6.70)	0.01
YSR internalizing problems (max. 80)	52.22 (13.45)	54.56 (15.47)	51.75 (13.02)	0.33
YSR externalizing problems (max. 80)	51.06 (12.78)	55.78 (14.78)	50.10 (12.18)	0.04
*ADHD symptoms*				
WR-SR attention difficulties (max. 30)	15.92 (5.14)	15.88 (5.21)	15.93 (5.14)	0.97
WR-SR hyperactivity (max. 15)	8.38 (3.22)	8.15 (3.27)	8.43 (3.22)	0.68
WR-SR temper (max. 15)	8.16 (3.47)	9.56 (3.23)	7.87 (3.46)	0.02
WR-SR affective lability (max. 20)	11.11 (3.78)	13.37 (4.14)	10.63 (3.54)	< 0.01
WR-SR emotional over-reactivity (max. 20)	10.05 (3.74)	12.74 (4.33)	9.48 (3.36)	< 0.01
WR-SR disorganization (max. 30)	14.79 (5.92)	18.37 (7.03)	14.03 (5.39)	< 0.01
WR-SR impulsivity (max. 25)	13.55 (4.71)	14.63 (5.85)	13.32 (4.43)	0.19
WR-SR oppositional symptoms (max. 45)	23.60 (6.97)	24.56 (8.60)	23.40 (6.59)	0.44
WR-SR academic problems (max. 20)	7.84 (3.34)	9.04 (4.11)	7.58 (3.11)	0.04
WR-SR social attitudes (max. 45)	20.79 (7.04)	24.11 (7.31)	20.08 (6.80)	< 0.01
WR-SR sum scores (max. 295)	134.20 (36.94)	150.41 (41.61)	130.76 (35.09)	0.01
*Intermittent explosive disorder*				
IED aggression score (max. 25)	12.81 (5.53)	13.33 (5.27)	12.70 (5.69)	0.59
Diagnosis of IED	56 (35.9%)	13 (48.2%)	43 (33.3%)	0.14

*CTQ* Childhood Trauma Questionnaire, *IED* intermittent explosive disorder, *WR-SR* Wender–Reimherr Self-Report Questionnaire, *YSR* Youth Self-Report

**Fig. 1:**
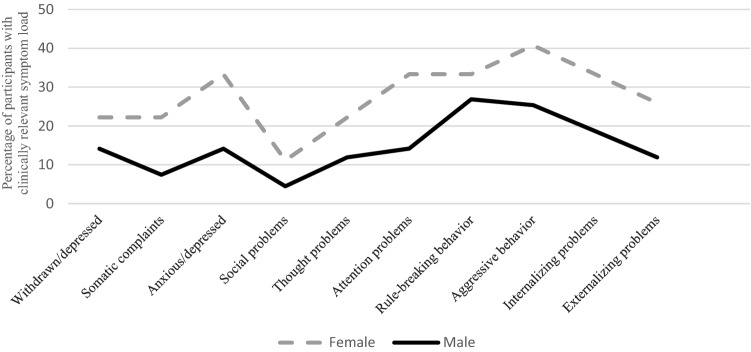
Percentages of participants above the cut-off for a clinically significant symptom load for each of the individual Youth Self-Report (YSR) subscales and the externalizing and internalizing broadband scales. Significant sex differences were found concerning somatic complaints, anxious/depressed and attention problems

Figure 1 gives an overview about the percentage of participants who were above the cut-off for a clinically significant symptom load within the single symptom scales of the YSR. More female than male participants showed a clinically significant symptom load in the following psychopathological symptom scales: somatic complaints, anxious/depressed and attention problems.

### Latent-class analysis on CTQ subscales

We compared latent class models with two–five classes according to the Akaike Information Criterion (AIC) and the Bayesian Information Criterion (BIC) [56, 57]. Smaller values indicate improved balance of parsimony and model fit. The 4-class model showed the smallest AIC; the 3-class model showed the smallest BIC (Table 2). Since the BIC has been proven superior for the identification of the number of latent classes in LCA over the AIC and because the 3-class model indicated classes of clear interpretability, the 3-class model was chosen for further analysis [58]. The relative entropy value of 0.89 indicated clear assignments of young offenders to respective latent classes or subtypes.

**Table 2:** Comparison of latent class model with two–five classes

Classes	Max. log-likelihood	*df*	AIC	BIC
2	− 394.0435	20	810.09	843.98
3	− 370.3416	14	774.68	827.06
4	− 360.8373	8	767.67	838.55
5	− 359.4151	2	776.83	866.79

*AIC* Akaike information criterion, *BIC* Bayesian information criterion, *df* degrees of freedom

**Fig. 2:**
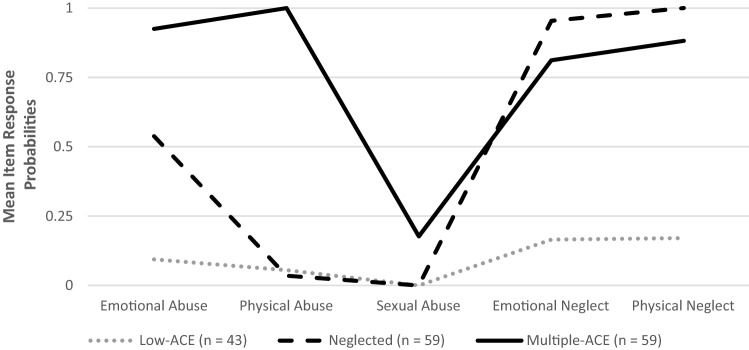
Adverse childhood experiences (ACE) subtypes based on latent class analysis (LCA) with Childhood Trauma Questionnaire (CTQ) subscales

Figure 2 displays the distinct ACE patterns for the three subtypes derived from the LCA. Based on the according item-response probabilities, we labeled the subtypes as (1) low-ACE subtype (*n* = 43, 26.7%), (2) mainly neglected subtype (*n* = 59, 36.7%), and (3) multiple-ACE subtype (*n* = 59, 36.7%). All subtypes differed significantly from each other on mean CTQ-poly-victimization scores, *F*(2, 151) = 434.84, *p* < 0.001. Offenders from the low-ACE subtype showed the lowest average CTQ-poly-victimization score (*M* = 0.43, SD 0.50), followed by the mainly neglected subtype (*M* = 2.52, SD 0.50) and the multiple-ACE subtype (*M* = 3.77, SD 0.63). The distribution of male and female offenders across subtypes was not significantly uneven, *χ*^*2*^(2) = 3.90, *p* = 0.143. MANOVA revealed age differences across subtypes, *F*(2, 151) = 3.73, *p* = 0.025, with offenders from the low-ACE subtype (*M* = 17.73 years, SD 1.58) being significantly younger than offenders from the mainly neglected subtype (*M* = 18.78 years, SD 2.04) and offenders from the multiple-ACE subtype (*M* = 18.77, SD 2.41). No differences were found regarding years of education, *F*(2, 151) = 2.62, *p* = 0.076, or type of offense, *χ*^*2*^(10) = 13.50, *p* = 0.184.

Results from the MANOVA concerning subtype differences on YSR symptom scales, WR-SR subscales, and IED overall aggression scores, as well as the *χ*^*2*^-statistic concerning respective group differences on IED diagnoses are presented in Table 3. Most YSR T-value scores differed between subtypes with young offenders from the low-ACE subtype showing lower scores than young offenders from the other subtypes. Only three WR-SR subscales differed with the multiple-ACE subtype showing the highest scores. No differences were found between subtypes on the two IED indicator variables.

**Table 3:** Average questionnaire scores divided by ACE subtypes

	Low ACE (*n *= 43)	Neglected (*n *= 59)	Multiple ACE (*n *= 59)	*p*
YSR withdrawn/depressed (max. 80)	51.28 (3.92)_a_	55.95 (7.41)_b_	57.41 (9.67)_b_	< 0.001
YSR somatic complaints (max. 80)	53.14 (6.07)	55.88 (8.20)	56.32 (6.98)	0.07
YSR anxious/depressed (max. 80)	52.79 (5.87)_a_	56.29 (8.40)_a,b_	58.85 (11.09)_b_	0.004
YSR social problems (max. 80)	50.93 (4.26)_a_	53.73 (5.60)_b_	53.78 (7.25)_b_	0.03
YSR thought problems (max. 80)	54.84 (4.64)_a_	56.44 (8.14)_a,b_	59.81 (10.60)_b_	0.01
YSR attention problems (max. 80)	51.93 (4.16)_a_	56.71 (8.94)_b_	57.10 (9.40)_b_	0.003
YSR rule-breaking behavior (max. 80)	52.19 (3.80)_a_	60.14 (10.20)_b_	60.76 (10.34)_b_	< 0.001
YSR aggressive behavior (max. 80)	51.26 (3.43)_a_	56.02 (9.07)_b_	55.02 (6.74)_b_	0.003
YSR internalizing problems (max. 80)	45.0 (10.58)_a_	53.66 (12.66)_b_	56.05 (14.23)_b_	< 0.001
YSR externalizing problems (max. 80)	43.91 (7.95)_a_	54.54 (13.17)_b_	52.78 (13.36)_b_	< 0.001
WR-SR attention difficulties (max. 30)	15.27 (4.46)	15.52 (5.29)	16.79 (5.40)	0.27
WR-SR hyperactivity (max. 15)	8.32 (3.34)	7.75 (3.38)	9.05 (2.87)	0.10
WR-SR temper (max. 15)	7.95 (3.26)	7.70 (3.93)	8.77 (3.11)	0.23
WR-SR affective lability (max. 20)	10.10 (3.50)_a_	10.63 (3.74)_a_	12.32 (3.75)_b_	< 0.01
WR-SR emotional over-reactivity (max. 20)	10.24 (3.83)_a_	8.69 (3.54)_b_	10.98 (3.66)_a_	0.01
WR-SR disorganization (max. 30)	13.56 (4.50)	14.61 (6.45)	15.86 (6.17)	0.16
WR-SR impulsivity (max. 25)	12.88 (4.50)	12.79 (4.57)	14.79 (4.82)	0.06
WR-SR oppositional symptoms (max. 45)	24.73 (6.23)	22.46 (8.11)	23.91 (6.15)	0.26
WR-SR academic problems (max. 20)	8.56 (2.80)	7.07 (3.24)	8.07 (3.68)	0.08
WR-SR social attitudes (max. 45)	19.54 (5.84)_a_	19.11 (7.52)_a_	23.33 (6.69)_b_	< 0.01
WR-SR sum scores (max. 295)	131.15 (32.72)_b_	126.59 (41.12)_b_	143.88 (33.80)_a_	0.04
IED aggression score (max. 25)	12.46 (5.98)	12.45 (5.99)	13.42 (4.71)	0.58
Diagnosis of IED	13 (31.71%)	17 (29.31%)	26 (45.61%)	0.15

Subscripts a, b represent group differences. Groups with different subscripts significantly differed from each other (*p *<0.05)

### Regression analyses

To analyze whether the person-centered ACE subtypes and/or the variable-centered CTQ-poly-victimization score statistically predicted mental health problems in the present sample, we further conducted two linear regression analyses for each YSR subscale as well as the WR-SR sum score and the IED total aggression score. In the first regression analysis, we used the ACE subtypes as predictor, and in the second regression analysis, the CTQ-poly-victimization score. Because sex differences had been found on mental health problem scales and age differences had been found across ACE subtypes, we included sex and age as covariates. Table 4 presents the results of the regression analyses. In sum, the CTQ-poly-victimization score was positively associated with elevated scores on each YSR subscale except for the social problems subscale. Similarly, both the neglected subtype and the multiple-ACE subtype were positively associated with elevated scores on most YSR subscales compared to the low-ACE subtype. The multiple-ACE subtype predicted YSR scores more frequently and mostly with stronger effect than the neglected subtype; however, regarding some subscales, the neglected subtype showed comparable or higher effect sizes. The WR-SR sum score was neither predicted by the CTQ-poly-victimization score, nor by ACE subtypes. Comparably, the IED total aggression score was not predicted by either indicator. Moreover, female sex appeared to significantly predict higher scores on most outcome variables.

**Table 4:** Results of linear regression analyses with CTQ-poly-victimization score and ACE subtypes as predictors, age and sex as control variables, and YSR subscales (*T* values), WR sum score, and IED total aggression score as outcomes

	YSR withdrawn/depressed				YSR somatic complaints				YSR anxious/depressed				YSR social problems			
	*B*	95% CI	*β*	*p*	*B*	95% CI	*β*	*p*	*B*	95% CI	*β*	*p*	*B*	95% CI	*β*	*p*
CTQ-poly	1.64	0.80, 2.48	0.29	0.01	1.00	0.20, 1.80	0.20	0.01	1.58	0.59, 2.57	0.25	0.01	0.54	− 0.12, 1.19	0.12	0.11
Age	0.20	− 0.37, 0.77	0.05	0.50	− 0.13	− 0.67, 0.41	− 0.04	0.63	0.18	− 0.49, 0.85	0.04	0.52	0.27	− 0.17, 0.71	0.09	0.23
Sex	− 1.98	− 5.18, 1.21	− 0.09	0.22	− 1.26	− 4.28, 1.77	− 0.06	0.41	− 1.75	− 5.50, 2.00	− 0.07	0.36	− 2.79	− 5.27, − 0.31	− 0.17	0.03
ACE subtypes																
Neglected	4.67	1.56, 7.79	0.28	0.01	3.05	0.10, 6.00	0.20	0.04	3.46	− 0.20, 7.12	0.18	0.06	2.75	0.35, 5.15	0.22	0.03
Multiple	5.85	2.74, 8.95	0.35	0.01	3.27	0.33, 6.21	0.22	0.03	5.80	2.15, 9.45	0.30	0.01	2.42	0.03, 4.81	0.19	0.05
Age	0.10	− 0.47, 0.69	0.03	0.71	− 0.18	− 0.74, 0.37	− 0.05	0.51	0.12	− 0.56, 0.80	0.02	0.73	0.19	− 0.25, 0.64	0.07	0.39
Sex	− 2.20	− 5.43, 1.04	− 0.10	0.18	− 1.48	− 4.54, 1.58	− 0.08	0.34	− 1.75	− 5.55, 2.04	− 0.07	0.36	− 2.97	− 5.46, − 0.48	− 0.18	0.02

	YSR thought problems				YSR attention problems				YSR rule-breaking behavior				YSR aggressive behavior			
	*B*	95% CI	*β*	*p*	*B*	95% CI	*β*	*p*	*B*	95% CI	*β*	*p*	*B*	95% CI	*β*	*p*
CTQ-poly	0.98	0.05, 1.91	0.16	0.04	1.49	0.60, 2.37	0.25	0.01	2.59	1.60, 3.58	0.38	0.01	1.29	0.53, 2.06	0.26	0.01
Age	0.45	− 0.18, 1.07	0.11	0.16	− 0.18	− 0.78, 0.42	− 0.05	0.55	− 0.21	− 0.88, 0.46	− 0.05	0.54	− 0.16	− 0.67, 0.36	− 0.05	0.56
Sex	− 3.73	− 7.25, − 0.22	− 0.16	0.04	− 4.16	− 7.51, − 0.81	− 0.19	0.02	− 2.31	− 6.06, 1.45	− 0.09	− 0.23	− 3.66	− 6.57, − 0.75	− 0.19	0.01
ACE subtypes																
Neglected	1.31	− 2.09, 4.74	0.07	0.45	5.41	2.16, 8.66	0.31	0.01	8.58	4.93, 12.23	0.43	0.01	5.33	2.51, 8.14	0.35	0.01
Multiple	4.27	0.88, 7.66	0.24	0.01	5.14	1.90, 8.38	0.30	0.01	8.75	5.12, 12.39	0.44	0.01	3.71	0.91, 6.52	0.25	0.01
Age	0.42	− 0.22, 1.05	0.10	0.19	− 0.29	− 0.90, 0.32	− 0.07	0.35	− 0.37	− 1.06, 0.31	− 0.08	0.28	− 0.25	− 0.78, 0.27	− 0.07	0.34
Sex	− 3.47	− 7.00, 0.06	− 0.15	0.05	− 4.58	− 7.95, − 1.21	− 0.20	0.01	− 2.94	− 6.73, 0.85	− 0.11	0.13	− 4.27	− 7.19, − 1.36	− 0.22	0.01

	YSR internalizing problems				YSR externalizing problems				WR sum score				IED aggression score			
	*B*	95% CI	*β*	*p*	*B*	95% CI	*β*	*p*	*B*	95% CI	*β*	*p*	*B*	95% CI	*β*	*p*
CTQ-poly	3.45	2.07, 4.84	0.37	0.01	2.79	1.46, 4.13	0.31	0.01	− 4.10	− 18.50, 10.3	− 0.05	0.58	− 0.31	− 2.63, 2.01	− 0.03	0.80
Age	0.35	− 0.59, 1.28	0.06	0.74	− 0.19	− 1.09, 0.71	− 0.03	0.68	2.32	− 0.31, 4.94	0.13	0.08	0.04	− 0.38, 0.46	0.01	0.86
Sex	− 1.84	− 7.08, 3.40	− 0.05	0.49	− 4.79	− 9.85, 0.27	− 0.14	0.06	− 18.79	− 33.41, − 4.17	− 0.19	0.01	− 0.60	− 2.94, 1.74	− 0.04	0.62
ACE subtypes																
Neglected	8.53	3.32, 13.74	0.31	0.01	11.50	6.65, 16.35	0.44	0.01	− 4.10	− 18.50, 10.30	− 0.05	0.58	− 0.31	− 2.63, 2.01	− 0.03	0.80
Multiple	10.62	5.43, 15.82	0.38	0.01	8.88	4.04, 13.71	0.34	0.01	11.17	− 3.16, 25.51	0.15	0.13	0.84	− 1.49, 3.16	0.07	0.48
Age	0.24	− 0.74, 1.21	0.04	0.48	− 0.42	− 1.33, 0.48	− 0.07	0.36	2.75	0.04, 5.45	0.16	0.05	0.08	− 0.35, 0.51	0.03	0.72
Sex	− 2.39	− 7.79, 3.01	− 0.07	0.38	− 5.95	− 10.98, − 0.93	− 0.18	0.02	− 17.90	− 32.81, − 2.99	− 0.18	0.02	− 0.56	− 2.94,1.83	− 0.04	0.65

The low-ACE subtype served as reference group for regression analysis involving ACE subtypes. Sex was coded as female = 1, male = 2

*ACE* adverse childhood experience, *CI* confidence interval, *CTQ* Childhood Trauma Questionnaire, *IED* intermittent explosive disorder, WR Wender–Reimherr Self-Report, *YSR* Youth Self-Report Questionnaire

## Discussion

The present study aimed at assessing the association between ACEs and a wide range of psychopathological symptoms including ADHD and IED in a sample of male and female adolescent and young adult offenders. Although there already exist previous studies addressing the relationship between ACEs and mental disorders, this connection has so far only seldomly been studied in young offenders, underscoring the relevance of our research project [38].

Comparable to previous studies, the young offenders reported about a higher rate of ACEs compared to adolescents from the general population [59, 60]. Interestingly, in the present study, female young offenders reported more frequently about childhood emotional, physical, and sexual abuse than male young offenders although studies with non-criminal juveniles suggested that only childhood sexual abuse occurs more frequently in girls than in boys [61, 62]. It could be possible that the rate of ACEs is underestimated in male individuals in the present study because reporting on ACEs could be associated with more feelings of shame and more stigmatization following disclosure in males than in females. Furthermore, it could be possible that female offenders may have to commit more severe offences to be placed in prison or a juvenile detention center than their male counterparts. ACEs are positively related to the frequency and severity of criminal offences thereby giving another possible explanation for the higher rate of ACEs in the female compared to the male participants [32].

Besides the risk-increasing effect of ACEs concerning criminal behaviors, they are also an important risk factor for the development of mental health problems in offending as well as non-offending juveniles [38]. This association could be supported by this study, as it was found that each additional type of ACE increased the risk to develop most of the examined mental health problems. This accounted for internalizing as well as externalizing mental health problems, thereby providing further empirical evidence for eminent criminological theories explaining the offending pathways of juvenile offenders [26, 32]. However, due to the cross-sectional nature of our study, it could also be possible that adolescents with more mental health problems during childhood, for example ADHD, conduct disorder or oppositional defiant disorder, are at greater risk of being maltreated during later childhood or adolescence. Furthermore, children of parents with mental health problems are at a greater risk to suffer from own mental disturbances and at the same time a mentally ill parent might be more likely to show abusive behavior against his or her children than a parent without mental health problems.

Nevertheless, mental health problems increase the risk for criminal offending and thus, identifying and treating the vulnerabilities (like maladaptive childhood experiences) that are closely associated with mental health disorders would additionally decrease the risk to show criminal behaviors [63, 64]. Although we did not address the association between the occurrence of mental health problems and criminal offending within the present study, we found a higher rate of psychopathological symptoms than what would have been suggested by previous research with non-criminal juveniles providing indirect evidence that mental health problems could be associated with offending [13]. However, it could also be possible that offending and subsequent incarceration results in the development of mental disorders. To explore the causal links between mental health problems and offending, longitudinal studies are needed.

We found that the female young offenders reported about more mental health disturbances measured with the YSR compared to their male counterparts, which is in line with the finding of more ACEs in the female than in the male young offenders. However, some findings need some closer inspection. Female offenders had higher average scores on the following YSR subscales: social problems, thought problems, attention problems, and aggressive behavior. At the same time, more female young offenders were above the cut-off for a clinically significant symptom load than their male counterparts on the following subscales: somatic complaints, anxiety/depression and again attention problems. These discrepancies suggest that even though female young offenders show more disturbances concerning social problems, thought problems, and aggressive behaviors, these sex differences remain on a sub-clinical level. However, previous research has shown that even mental health problems under the diagnostic threshold can cause relevant impairments in daily life, for example criminal behaviors [65]. Only concerning attention problems, the female offenders showed more disturbances than the male offenders irrespective of the analytic approach pointing out that female offenders show such problems on a sub-clinical as well as on a clinical level. Thus, it can be concluded that attention problems or ADHD symptomatology is especially relevant in young female offenders.

In addition to earlier findings supporting a dose-dependent relationship between the number of different ACEs and the probability of various mental health disturbances, the results of our LCA suggested that not only the quantity but also the quality of ACEs is relevant for the occurrence of mental health problems. We found three different subtypes based on ACEs: one subtype low on ACEs, one subtype with mainly neglectful experiences, and a third subtype with multiple ACEs. Although individuals who reported multiple ACEs were at highest risk of showing mental disturbances, young offenders from the neglectful subtype showed similar and even stronger associations with specific outcome variables such as social problems and aggressive behaviors. These findings (1) support the previously highlighted role of neglectful experiences in young and adult offending populations and (2) underscore the importance to focus not only on the sum of ACEs when associations with specific outcomes are being investigated but also on specific person-centered ACE patterns [24, 25, 41].

High prevalence rates of ADHD in samples of young offenders were reported in previous studies [66, 67]. Comparably, the average WR-SR scores were considerably higher than the scores found in individuals from the general population [49, 51]. Coming back to the theoretical considerations of Moffitt, previous studies reported a higher prevalence of ADHD in life-course persistent compared to adolescent-limited offenders [68]. Furthermore, in congruence with samples from the general population, cumulated ACEs were significantly associated with ADHD symptomatology [18, 69, 70], which is represented by higher WR-SR sum scores in young offenders from the multiple-ACE subgroup compared to young offenders from the mainly neglected and the low-ACE subgroups. Interestingly though, the three groups did not differ concerning the core ADHD symptoms (attention difficulties, hyperactivity, and impulsivity) but rather concerning ADHD-associated symptom clusters, e.g., affective lability, emotional over-reactivity, and social attitudes. Previous studies found that deficits in emotion regulation correlated with physical and verbal aggression, and other rule-breaking behaviors [71, 72]. It was suggested that impairments in emotional regulation could lead to more intense reactions to certain emotional stimuli causing impulsive aggressive and reactive violent behaviors explaining how problems in emotion regulation could lead to offending [9, 73]. Somewhat limiting the plausibility of this suggestion is the finding that in our regression analyses, the YSR attention problems subscale was significantly predicted by the ACE poly-victimization score pointing out that not only ADHD-related symptom clusters are associated with more ACEs but also ADHD core symptoms. Why results of the regression analyses differ concerning the YSR attention problems subscale and the WR-SR subscales, although one would expect that both scales measure somewhat comparable constructs, remains unclear. This finding could, however, speak against the congruent validity of both scales.

Finally, the present study is among the first to assess the prevalence of IED in a sample of young offenders. More than a third of the present adolescent offenders could be diagnosed with an IED clearly exceeding the rates of previous studies with community samples [74]. An IED diagnosis increased the risk to be convicted for a violent offence almost 10-times in a sample of more than 10,000 adolescents from the US, underscoring the importance of an IED diagnosis for criminal risk assessment [75]. Adding some more important information to the current state of research, we found that an IED can be found at a comparable rate in male and female adolescent offenders. Our data did not support associations between IED and ACEs which was surprising as previous research in non-offender samples has found respective associations [20, 76].

Our results are limited because they are solely based on self-report measures. This could have led to an underestimation of the actual frequency of ACEs because for many individuals, disclosure of ACEs could be associated with feelings of shame and stigmatization. The same accounts for psychopathological symptoms, especially those of the internalizing problem scales. Thus, future studies should use different forms of gathering the relevant data, for example, self-report questionnaires, diagnostic interviews and informant data to get a more holistic view. The present study was the first that used a German translation of the newly developed IED screening questionnaire for the assessment of an IED diagnosis and was, to our knowledge, the first study conducted in a Western country that assessed the prevalence of IED in young offenders. Although satisfying psychometric properties of the questionnaire were found in the present study, clearly more studies assessing the questionnaire’s reliability and validity are needed before it can be used on a regular basis. Furthermore, our results are somewhat limited because of the small sample size concerning our female adolescent offenders. Clearly, more studies are needed assessing the criminological and personal characteristics and needs of female young offenders. Furthermore, future research should evaluate the effectiveness of therapeutic interventions addressing the mental health needs of young offenders. A recent meta-analysis revealed that psychotherapeutic interventions are effective in reducing violent offending in adults; however, it is still unknown whether or not the same accounts for young offenders at the beginning of their criminal careers [77]. It would be worthwhile to investigate whether or not such interventions only decrease the mental health burden or also the rate of recidivism in adolescent offenders with a mental health problem. A further limitation concerns the cross-sectional study design, which prevents causal interpretations. Thus, the findings of the current study should be replicated in a longitudinal design, opening better insights in possible pathways from ACEs to psychopathology/criminal behavior.

As stated in our opening sentence, young offenders are an especially vulnerable group needing increased attention. Most of the young people who have come into contact with the justice system are still at the very beginning of their criminal careers making supervisory and therapeutic interventions especially meaningful and worthwhile. This is particularly the case for the present sample who were either first-time offenders or those with so far only rather minor offences. Because of the limited financial and personal resources usually allocated to the supervision and treatment of juvenile offenders, the used interventions should be based on and guided by the current state of research. Not only did we find a considerable prevalence of ACEs, but also that ACEs were linked to a higher rate of mental health disturbances. Thus, it can be suggested that ACEs should be assessed and addressed on a more regular basis.
